# Muscle cells of sporadic amyotrophic lateral sclerosis patients secrete neurotoxic vesicles

**DOI:** 10.1002/jcsm.12945

**Published:** 2022-02-22

**Authors:** Laura Le Gall, William J. Duddy, Cecile Martinat, Virginie Mariot, Owen Connolly, Vanessa Milla, Ekene Anakor, Zamalou G. Ouandaogo, Stephanie Millecamps, Jeanne Lainé, Udaya Geetha Vijayakumar, Susan Knoblach, Cedric Raoul, Olivier Lucas, Jean Philippe Loeffler, Peter Bede, Anthony Behin, Helene Blasco, Gaelle Bruneteau, Maria Del Mar Amador, David Devos, Alexandre Henriques, Adele Hesters, Lucette Lacomblez, Pascal Laforet, Timothee Langlet, Pascal Leblanc, Nadine Le Forestier, Thierry Maisonobe, Vincent Meininger, Laura Robelin, Francois Salachas, Tanya Stojkovic, Giorgia Querin, Julie Dumonceaux, Gillian Butler Browne, Jose‐Luis González De Aguilar, Stephanie Duguez, Pierre Francois Pradat

**Affiliations:** ^1^ Northern Ireland Center for Stratified Medicine Biomedical Sciences Research Institute Londonderry UK; ^2^ Sorbonne Université, Institut National de la Santé et de la Recherche Médicale, Association Institut de Myologie, Centre de Recherche en Myologie Paris France; ^3^ I‐Stem, INSERM/UEVE UMR 861, I‐STEM, AFM Paris France; ^4^ NIHR Biomedical Research Centre University College London, Great Ormond Street Institute of Child Health and Great Ormond Street Hospital NHS Trust London UK; ^5^ Inserm U1127 CNRS UMR7225, Sorbonne Universités, UPMC Univ Paris France; ^6^ Genetic Medicine, Children's National Medical Center George Washington University Washington DC USA; ^7^ The Neuroscience Institute of Montpellier, Inserm UMR1051 Univ Montpellier, Saint Eloi Hospital Montpellier France; ^8^ Mécanismes Centraux et Périphériques de la Neurodégénérescence Université de Strasbourg, INSERM UMR_S 1118 Strasbourg France; ^9^ Computational Neuroimaging Group Academic Unit of Neurology Trinity College Dublin Dublin Ireland; ^10^ CNRS, INSERM, Laboratoire d'Imagerie Biomédicale Sorbonne Université Paris France; ^11^ APHP, Département de Neurologie Hôpital Pitié‐Salpêtrière, Centre référent SLA Paris France; ^12^ APHP, Centre de Référence des Maladies Neuromusculaires Nord/Est/Ile de France, Institut de Myologie Hôpital Pitié‐Salpêtrière Paris France; ^13^ Laboratoire de Biochimie et Biologie Moléculaire Hôpital Bretonneau, CHRU de Tours Tours France; ^14^ INSERM U1171, Pharmacologie Médicale & Neurologie Université, Faculté de Médecine CHU de Lille Lille France; ^15^ Département de Neurologie, Centre de Référence Maladies Neuromusculaires Paris‐Est Hôpital Raymond‐Poincaré Garches France; ^16^ Laboratory of Molecular Biology of the Cell Ecole Normale Supérieure de Lyon Lyon France; ^17^ Hôpital des Peupliers Ramsay Générale de Santé Paris France

**Keywords:** Secreted vesicles, Cell–cell communication, MND, sporadic ALS

## Abstract

**Background:**

The cause of the motor neuron (MN) death that drives terminal pathology in amyotrophic lateral sclerosis (ALS) remains unknown, and it is thought that the cellular environment of the MN may play a key role in MN survival. Several lines of evidence implicate vesicles in ALS, including that extracellular vesicles may carry toxic elements from astrocytes towards MNs, and that pathological proteins have been identified in circulating extracellular vesicles of sporadic ALS patients. Because MN degeneration at the neuromuscular junction is a feature of ALS, and muscle is a vesicle‐secretory tissue, we hypothesized that muscle vesicles may be involved in ALS pathology.

**Methods:**

Sporadic ALS patients were confirmed to be ALS according to El Escorial criteria and were genotyped to test for classic gene mutations associated with ALS, and physical function was assessed using the ALSFRS‐R score. Muscle biopsies of either mildly affected deltoids of ALS patients (*n* = 27) or deltoids of aged‐matched healthy subjects (*n* = 30) were used for extraction of muscle stem cells, to perform immunohistology, or for electron microscopy. Muscle stem cells were characterized by immunostaining, RT‐qPCR, and transcriptomic analysis. Secreted muscle vesicles were characterized by proteomic analysis, Western blot, NanoSight, and electron microscopy. The effects of muscle vesicles isolated from the culture medium of ALS and healthy myotubes were tested on healthy human‐derived iPSC MNs and on healthy human myotubes, with untreated cells used as controls.

**Results:**

An accumulation of multivesicular bodies was observed in muscle biopsies of sporadic ALS patients by immunostaining and electron microscopy. Study of muscle biopsies and biopsy‐derived denervation‐naïve differentiated muscle stem cells (myotubes) revealed a consistent disease signature in ALS myotubes, including intracellular accumulation of exosome‐like vesicles and disruption of RNA‐processing. Compared with vesicles from healthy control myotubes, when administered to healthy MNs the vesicles of ALS myotubes induced shortened, less branched neurites, cell death, and disrupted localization of RNA and RNA‐processing proteins. The RNA‐processing protein FUS and a majority of its binding partners were present in ALS muscle vesicles, and toxicity was dependent on the expression level of FUS in recipient cells. Toxicity to recipient MNs was abolished by anti‐CD63 immuno‐blocking of vesicle uptake.

**Conclusions:**

ALS muscle vesicles are shown to be toxic to MNs, which establishes the skeletal muscle as a potential source of vesicle‐mediated toxicity in ALS.

## Introduction

Amyotrophic lateral sclerosis (ALS) is a fatal adult‐onset motor neuron disorder affecting 3–5/100 000 individuals per year.^1^ The cause of pathology is likely complex with onset resulting from some combination of genetic mutations, DNA damage, environmental risk factors, viral infections, or other factors, leading to diverse cellular dysfunction such as glutamate‐mediated excitotoxicity, abnormal protein aggregation, and mitochondrial disorganization and dysfunction contributing to oxidative stress (refer to Vijayakumar *et al*.^2^ and Le Gall *et al*.^3^ for review).

Not only motor neurons (MN) are affected in ALS, but also glial cells, muscle fibres,^4^ and immune cells,^5^ each of which may participate actively in ALS onset and progression. In a FUS murine model of motor neurone disease, where the FUS mutation is expressed in all tissues except the MN, motor deficits still appear at a late stage of the disease.^6^ In addition, when the human mutated SOD1(hSOD1) is selectively knocked‐down in MN and astrocytes of newborn transgenic hSOD1 mice, their life expectancy is prolonged by only 65–70 days, and there is still significant astrogliosis and microglial activation.^7^ These studies support the potential role of ‘non‐cell autonomous’ MN death in ALS, which could involve multiple cell types.^8^

Numerous cell and tissue types, including skeletal muscle, can secrete exosomes and other types of vesicle.^9^, ^10^ Such extracellular vesicles may be involved in cell–cell communication in the central nervous system,^11^, ^12^ where they can carry out intercellular transport of functional proteins, mRNA, miRNA, and lipids and may have key roles in spreading of proteinopathies^11^, ^12^ or neurotoxic elements.^13^ For instance, astrocytes extracted from the SOD1 murine model of ALS secrete exosomes that contain hSOD1 and propagate this toxic protein to neighbouring MNs.^14^ The present study sought to determine whether vesicle secretion is affected in muscle cells of ALS subjects, and whether ALS muscle vesicles (MuVs) could be toxic when taken up by recipient motor neurons.

## Material and methods

### Participants and ethical approvals

An open biopsy was performed on deltoid muscles of 27 ALS patients with probable or definite ALS according to the revised El Escorial criteria,^15^ (who attended the Motor Neuron Diseases Center (Pitié Salpétrière, Paris). To guarantee a consistent quality of muscle tissue, deltoid samples were taken using the procedure that is consistently and routinely followed for all muscle sampling at the centre (ALS Referral Center, Pitie Salpetriere, Sorbonne University). The affectedness of each patient's deltoid muscle was assessed directly by the Manual Muscle Testing scale, against which all muscles sampled were determined to be at least at Level 3 indicating muscle movement through full range of motion against gravity. Genetic analyses were carried out on DNA extracted from blood samples for all ALS patients to screen several ALS‐related genes: the *C9orf72* hexanucleotide repeat expansion (using gene scan and repeat primed PCR procedures described in Millecamps *et al*.^16^), *ATXN2* repeat length,^17^ and the coding regions of *SOD1*, *TARDBP*, *FUS*, *UBQLN2*, and *TBK1* (sequences of the used primers are available upon request). Thirty deltoid muscle biopsies from healthy age‐matched and gender‐matched subjects were obtained from the BTR (Bank of Tissues for Research, a partner in the EU network EuroBioBank) in accordance with European recommendations and French legislation. The main demographic, clinical, and genetic characteristics of the subjects are indicated in *Table* 1.

**Table 1:** Summary of the samples used from different subjects for each type of experiment

											Cell culture						
ALS/healthy	57 subjects	Age at time of biopsy (years)	Gender	ALS/FRS	Genetics	Muscle testing	Site of onset 1 = upper limb 2 = lower limb; 3 = bulbar; 4 = respiratory	Disease duration (months)	Histology	Electron microscopy	Transcriptome	Immunostaining cell culture	RT‐qPCR	MuVs for proteomic analysis	MuVs for western blot	MuVs treatment MN	MuVs treatment myotubes
ALS	27 ALS patients 8 F, 19 M 57.44 ± 9.47	50–59	F	38	*—*	130	1	13	x								
ALS		60–69	M	45	*—*	146	2	25.2			x				x		x
ALS		50–59	M	41	*—*	140	2	26.7			x						x
ALS		50–59	F	39	*C9orf72*	146	3	16.5				x			x	x	
ALS		50–59	F	28	*C9orf72*	119	3	6	x			x	x		x		x
ALS		60–69	M	38	*ATXN2*	130	2	11.3			x		x			x	
ALS		60–69	M	40	*—*	130	1	39.3			x		x		x		x
ALS		70–79	M	37	*—*	126	3	48.3			x		x		x		x
ALS		60–69	M	33	*—*	123	1	53.4	x		x		x		x	x	
ALS		70–79	M	32	*—*	104	1	28				x		x	x		
ALS		60–69	F	30	*—*	97	1	71.6				x			x		
ALS		40–49	M	31	*—*	90	1	70							x		
ALS		50–59	F	23	*—*	103	3	33							x	x	
ALS		60–69	M	40	*—*	97	1	11				x			x		
ALS		60–69	M	38	*—*	88	1	52				x			x		
ALS		50–59	M	31	*—*	94	2	40							x	x	x
ALS		50–59	M	37	*—*	No data	0	101		x							
ALS		30–39	F	32	*—*	No data	0	85		x							
ALS		50–59	F	43	*—*	135	1	33.6	x			x			x		
ALS		40–49	M	41	*—*	111	2	44.1				x	x		x		x
ALS		50–59	M	40	*—*	130	2	17.4				x	x		x		
ALS		50–59	M	37	*C9orf72*	145	3	20.6				x		x	x		x
ALS		60–69	M	41	*—*	147	2	28.7				x			x	x	
ALS		50–59	M	36	*—*	139	2	12	x								
ALS		60–69	M	43	*—*	149	1	12	x			x			x	x	
ALS		30–39	M	41	*—*	144	1	12				x		x	x		
ALS		50–59	F	40	*—*	98	3	14		x							
Healthy	30 healthy 10 F; 20M 51.08 ± 18.40	60–69	M	—	*—*	—	—	—	x								
Healthy		70–79	M	—	*—*	—	—	—	x								
Healthy		50–59	F	—	*—*	—	—	—	x								
Healthy		60–69	F	—	*—*	—	—	—	x								
Healthy		50–59	M	—	*—*	—	—	—	x								
Healthy		50–59	F	—	*—*	—	—	—				x			x	x	
Healthy		40–49	F	—	*—*	—	—	—				x	x		x		
Healthy		50–59	M	—	*—*	—	—	—							x	x	
Healthy		40–49	M	—	*—*	—	—	—			x				x		
Healthy		50–59	M	—	*—*	—	—	—			x		x		x	x	
Healthy		50–59	M	—	*—*	—	—	—			x	x			x		x
Healthy		50–59	F	—	*—*	—	—	—				x	x	x	x		x
Healthy		40–49	F	—	*—*	—	—	—					x		x		x
Healthy		50–59	M	—	*—*	—	—	—			x	x	x		x		x
Healthy		70–79	M	—	*—*	—	—	—			x		x		x	x	
Healthy		20–29	M	—	*—*	—	—	—			x						
Healthy		20–29	M	—	*—*	—	—	—									x
Healthy		50–59	M	—	*—*	—	—	—		x							
Healthy		60–69	F	—	*—*	—	—	—		x							
Healthy		60–69	M	—	*—*	—	—	—		x							
Healthy		20–29	M	—	*—*	—	—	—				x			x	x	x
Healthy		30–39	M	—	*—*	—	—	—				x		x	x	x	
Healthy		20–29	M	—	*—*	—	—	—				x		x	x	x	x
Healthy		70–79	M	—	*—*	—	—	—				x					
Healthy		70–79	F	—	*—*	—	—	—				x					
Healthy		70–79	M	—	*—*	—	—	—				x					
Healthy		80–89	M	—	*—*	—	—	—				x					
Healthy		20–29	M	—	*—*	—	—	—				x			x		
Healthy		60–69	F	—	*—*	—	—	—				x			x		
Healthy		40–49	F	—	*—*	—	—	—				x			x		

Subject age at time of biopsy is indicated in the column ‘Age’. All amyotrophic lateral sclerosis (ALS) patients were confirmed to be ALS according to El Escorial. The ALSFRS‐R and Muscle Testing results measured at the time of the biopsies are given. The manual muscle testing score is the sum of 30 measurements (15 muscle groups assessed once on each side of the body), each scored from 0, representing total paralysis, to 5, representing normal strength, according to the Medical Research Council Score.

The protocols (NCT01984957) and (NCT02360891) were approved by the local Ethical Committee and all subjects signed an informed consent in accordance with institutional guidelines.

#### Muscle stem cell extraction and culture

Briefly, muscles biopsies were dissociated mechanically as previously described in Bigot *et al*.^18^ and plated in proliferation medium [1 volume of M199, 4 volumes of Dulbecco's modified Eagle's medium (DMEM), 20% foetal bovine serum (v:v), 25 μg mL^−1^ Fetuin, 0.5 ng mL^−1^ bFGF, 5 ng mL^−1^ EGF, 5 μg mL^−1^ insulin]. The myogenic cell population was enriched using CD56 magnetic beads, and for their myogenicity using anti‐desmin antibodies as described before.^18^ A minimum of 80% of the cell population was positive for desmin. After rinsing three times the proliferative myoblasts with phosphate‐buffered saline (PBS), and three times with DMEM to remove any FBS residual, the human muscle stem cells were differentiated into myotubes by culturing them in DMEM for 3 days. All cell cultures were regularly checked every 3 weeks for mycoplasma test. All experiments were conducted in cells at less than 21 divisions to avoid potential cellular senescence, and thus experimental artefacts.

#### Muscle vesicle extraction from culture medium

For each replicate, 7.5 × 10^6^ primary myoblasts with less than 21 divisions were plated in 225 cm^2^ Flask. After 24 h, the cells were rinsed 6 times in DMEM, then differentiated into myotubes for 3 days in DMEM. All cell cultures were checked and negative for mycoplasma. Muscle vesicles were extracted from conditioned media as previously described for large‐scale isolation protocol.^19^, ^20^ Briefly the conditioned media were centrifuged at 260 g for 10 min at room temperature, then at 4000 *g* for 20 min at 4°C to remove any dead cells and cell debris, and finally at 20 000 *g* for 1 h at 4°C to remove microparticles. The subsequent supernatant was then filtered through 0.22 μm filter to remove any microparticles leftover. The filtered medium was then mixed with total exosome isolation reagent (Life technologies™; 2:1, v:v), incubated overnight at 4°C, and then centrifuged at 10 000 *g* at 4°C for 1 h. The supernatant was discarded, and the pellet containing the MuVs was resuspended and rinsed three times in PBS using 100 K MWCO column. The 100 μL MuVs suspensions were kept at −80°C until needed. MuVs were either used for treating cell cultures (iPSC‐derived motor neurons, or myotubes; treatments being always compared with untreated cells) or for protein content characterization. MuVs protein was extracted using 8 M Urea or NuPAGE buffer and quantified using BCA kit. Refer to *Figure* S2A and supporting information. The vesicles extracted using this protocol floated at similar density than when extracted by classic ultracentrifugation, 1.15–1.19 g mL^−1^, with a better yield was observed.^20^ Vesicles were positive for CD63, CD81, CD82, Flotillin, ALIX, and negative for calnexin (*Figure* 2 and Le Gall *et al*.^20^).

### NanoSight

The MuVs pellets were resuspended in 100 μL of filtered PBS. The MuV suspension was then diluted 10× in PBS. Size and distribution of MuV secreted by primary muscle cells were evaluated by a NanoSight LM10 instrument (NanoSight) equipped with NTA analytic software (version 2.3 build 2.3.5.0033.7‐Beta7). Samples were assessed three times as previously described^21^, ^22^ at temperature set to 22.5°C. The minimum particle size, track length, and blur were set to ‘automatic’.

### Muscle vesicles labelling

The MuVs were labelled using PKH26 kit (Sigma‐Aldrich®). Briefly, after adding 100 μL of Diluent C to the MuV suspension, 100 μL of 4 μM PKH26 solution were added to the sample. After 5 min of incubation, 1 mL PBS was added, and the MuVs were washed using a 100 K concentrators, 15 000 *g* at 4°C for 10 min. The MuVs were washed three times in PBS using the 100 K concentrators before being mixed with the cell media for treatment. All cell cultures treated with MuVs were compared and normalized to untreated cell cultures.

### Muscle vesicles added to iPSC motor neurons

The hiPSC‐derived motor neurons were obtained as previously described.^23^ There were 3000 MN progenitors differentiated for 9 days then plated in poly‐L‐ornithine (SIGMA) Laminin (Life Technologies) coated 384 well plates in differentiation medium N2B27 (DMEM F12, Neurobasal v:v, supplemented with N2, B27, Pen‐Strep, βMercaptoethanol 0,1%, Glutamax) supplemented with 100 nM Rock Inhibitor (RI), 100 nM Retinoic Acid (RA), 500 nM SAG, 100 nM DAPT, 10 ng/mL BDNF and Laminin. The medium was replaced at 11 days of differentiation with N2B27 supplemented with 200 nM RA, 1 μM SAG, 20 ng/mL BDNF, and 200 nM DAPT and again at 14 days of differentiation with N2B27 supplemented with 200 nM RA, 20 ng/mL GDNF, 20 ng/mL BDNF, and 200 nM DAPT. Sixteen‐days differentiated motor neurons were either treated with ALS MuVs or healthy MuVs resuspended in N2B27 differentiation medium supplemented with 20 ng/mL GDNF and 20 ng/mL BDNF. Cultures were fixed with 4% formaldehyde at 3 days of treatment. The MN were labelled for Tuj1, Islet 1/2 and analysed as described in the immuno‐labelling section. MN loss was normalized to untreated MN cultures. All cell culture treated with MuVs were compared and normalized to untreated cell culture.

### Muscle vesicles pre‐treatment with CD63 antibody

After labelling 0.5 μg MuVs with PKH26 as described earlier, the MuV suspension was incubated for 2 h at RT with 0.5 μg of CD63 antibody (TS63, Life Technologies) and then added to the culture medium of hiPSC‐derived motor neurons as described in the paragraph ‘Muscle vesicles added to iPSC motor neurons’. All cell cultures treated with MuVs were compared and normalized with untreated cell cultures.

#### Muscle vesicles added to healthy human muscle cells

Labelled MuVs were added to the differentiation medium of 200 000 control cells cultured in Ibidi 35 mm μ‐Dishes. MuVs absorption occurred during the first 3 days of differentiation. The myotubes were then rinsed three times with PBS, and fresh DMEM was added to the petri‐dishes. The cells were fixed with 3.6% formaldehyde for 15 min at room temperature at Day 3 or Day 7 of differentiation, then washed three times in PBS and stored at 4°C until subsequent analysis:

- *Myonuclear domain*—The myotubes were fixed and stained for DAPI and MF20 as described earlier. The myonuclear domain was calculated using the following formula: $\text{Myonuclear domain}=\frac{\left(\Sigma \;\mathrm{MF}20\;\text{area}\right)\;}{\left(\Sigma \;\text{nuclei in myotubes}\right)\;}$.
- *Stress blebbing*—Blebs were counted on live images at 4, 24, 48, 72, 96, and 168 h.
- *Cell death inducing an increase in H2Ax expression level*—The myotubes were fixed and stained for DAPI and H2AX as described earlier. To measure H2AX signal per myonucleus, the total area of H2AX signal was divided by the total number of myonuclei.
- *Cell loss*—The total number of nuclei were counted in each field using ImageJ 1.37v and summed for each well.
- *Different doses tested*—4 and 8 μg of MuVs were added to the culture medium of healthy myotubes. H2AX signal per myonucleus was assessed as described earlier.

All cell cultures treated with MuVs were compared and normalized with untreated cell cultures.

#### RNA extraction

Purified muscle stem cells were differentiated for 3 days into myotubes. RNA from muscle cells was extracted as described in Bigot *et al*.^18^ The quality of RNA samples was assessed with Agilent 2100 Bioanalyzer (Agilent Technologies Inc., Santa Clara, CA, USA).

#### Gene expression profiling

- *mRNA gene expression profiling—*Aliquots of high‐quality total RNA from each sample (ALS *n* = 6 and healthy *n* = 6, muscle stem cells) were used for mRNA expression profiling using GeneChip Human Exon 1.0 ST arrays (Affymetrix) as previously described.^24^
- *Analysis of gene expression data*—refer to the supporting information.

#### Immunolabelling

- *Immunocytochemistry*: 200 000 cells or 100 000 cells were respectively plated on u‐dish 35 mm high ibidiTreat or 4 wells plate ibidiTreat (ibidi®) in proliferative medium. The following day, muscle stem cells were washed with PBS and myogenic differentiation was induced by cultivating the cells in DMEM only. Muscle cells were fixed at 3 days of differentiation using 4% formaldehyde. The cells were permeabilized, blocked and stained as described.^9^
- *Immunohistology*: 8 μm muscle transverse sections were cut from human biopsies on a cryostat microtome at −20°C, permeabilized, blocked, and stained as previously described.^18^

Primary antibodies used are listed in the table below and the secondary antibodies used were goat anti‐mouse IgG1 or anti‐mouse IgG2a, or mouse IgG2b or anti‐rabbit tagged with AlexaFluor 355 or AlexaFluor 488 or AlexaFluor 555 or AlexaFluor 594 or AlexaFluor 647 (1:400, Invitrogen™). The slides were washed, counter‐stained with 1 μg mL^−1^ DAPI for 1 min, rinsed two times and mounted with ibidi mounting medium (ibidi®).

Ten to twenty non‐overlapping pictures were acquired in a line along the diameter of the slide with an Olympus IX70, and an Olympus UPlan FI 10×/0.30 Ph1 and an Olympus BX60 objectives equipped with a Photomatics CoolSNAP™ HQ camera. For the human muscle sections, pictures were taken with an Olympus LCPlan FI 40×/0.60 Ph2 objective. Images were acquired using Zeiss software and analysed using either Fiji or ImageJ 1.37v.

**Table:** 

Antibody	Clone	Manufacturer, Catalogue number	Species raised, Isotype	Dilution	Application
ALIX	3A9	Invitrogen, MA1‐83977	Monoclonal, IgG1	1:500	WB
Calnexin	AF18	Life Technologies, MA3‐027	Monoclonal, Mouse IgG1	1:100	WB
Caprin (GPIP137)		Life Technologies, PA5‐29514	Polyclonal, Rabbit IgG	1:200	ICC
CD63		BD Pharmingen	Monoclonal, Mouse IgG1	1:200	IHC, ICC
CD63	TS63	Life Technologies, 10628D	Monoclonal, Mouse IgG1	2 μg/mL	WB
CD81	M38	Invitrogen, 10630D	Monoclonal, Mouse IgG1	1:200	WB
CD82		Invitrogen, PA5‐27233	Polyclonal, Rabbit	1:200	EM
Dystrophin	3B7	DSHB, MANDYS1	Monoclonal, Mouse IgG2a	1:200	ICC
Flotillin		Life Technologies, PA5‐18053	Polyclonal, Goat IgG	0.3 μg/mL	WB
FUS		Bethyl, A300‐302A	Polyclonal, Rabbit IgG	1:2000	ICC
FUS		Life Technologies, PA5‐23696/PA5‐52610	Polyclonal, Rabbit IgG	0.4 μg/mL	WB, ICC
H2AX		Cell Signalling, 763J	Monoclonal, Rabbit IgG	1:50	ICC
Isl1/2	39.4D5	DSHB	Monoclonal, Mouse IgG2b	1:100	ICC
Myosin Heavy Chain	MF20	DSHB	Monoclonal, Mouse IgG2b	10 μg/mL	ICC
RPL5		Life Technologies, PA5‐26269/PA5‐27539	Polyclonal, Rabbit IgG	1:500	ICC
RPL5		Cell Signaling, 14568S	Polyclonal, Rabbit IgG	1:1000	WB
SOD1		Bethyl, A303‐812A	Polyclonal, Rabbit	0.1 μg/mL	WB
TDP43		Invitrogen, PA5‐17011	Polyclonal, Rabbit	1: 1000	WB
Tsg101	C‐2	Santa Cruz, sc‐7964	Monoclonal, Mouse IgG2a	1:200	ICC
Anti‐tubulin antibody, beta III isoform	TUJ1	Millipore, MAB‐1637	Monoclonal, Mouse IgG1	1:250	ICC
Skeletal alpha‐actin		Invitrogen	Polyclonal, Rabbit	1:1000	WB

### Electron microscopy

- *Electron Microscopy for extracted MuVs—*Purified vesicles were fixed in 2% paraformaldehyde and were counterstained with uranyl and lead citrate and analysed as previously described.^10^
- *Electron microscopy for human myotubes*—Human myotubes were plated on plastic (Thermanox, Nalge Nunc, Rochester, NY, USA) coverslips and fixed in 2.5% glutaraldehyde in 0.1 M phosphate buffer (*v*/v), pH 7.4 and further post‐fixed in 2% OsO4 (*w*/*v*). They were gradually dehydrated in acetone including a 1% uranyl en‐bloc staining step in 70% acetone (*w*/*v*), and embedded in Epon resin (EMS, Fort Washington, PA, USA). Ultrathin sections were counterstained with uranyl and lead citrate. Observations were made using a CM120 transmission electron microscope (Philips, Eindhoven, the Netherlands) at 80 kV and images recorded with a Morada digital camera (Olympus Soft Imaging Solutions GmbH, Münster, Germany).
- *Electron microscopy for human muscle biopsies*—Human muscles biopsies were fixed in 2% glutaraldehyde, 2% paraformaldehyde, 0.1 M phosphate buffer. After abundant washes and 2% OsO4 post‐fixation samples were dehydrated at 4°C in graded acetone including a 1% uranyl acetate in 70° acetone step and were finally embedded in Epon resin. Thin (70 nm) sections were stained with uranyl acetate and lead citrate, observed using a Philips CM120 electron microscope (Philips Electronics NV) and photographed with a digital SIS Morada camera.

### Analysis of gene expression data

Expression data were uploaded to the GEO repository at accession number GSE122261. Raw data (.CEL Intensity files) were processed using R/Bioconductor packages. Briefly, sample quality was verified by assessment of MA plots, normalized unscaled standard error (NUSE; all samples had median <1.1), and relative log expression (RLE; all sample had divergence <0.2), using the oligo package. Background‐corrected normalized log2‐transformed probe set signal intensities were obtained by robust multi‐array averaging (RMA) using default settings. Affycoretools and huex10stprobeset.db were used to annotate probeset IDs. For gene‐level analysis, probesets were retained if they had expression level >log_2_50 in at least 15% of samples. A design matrix (~0 + condition) was created, to which Limma was used to fit a linear model to the normalized expression values, and differentially expressed genes were identified using Limma's empirical Bayes method.

For enrichment mapping, the GSEA tool (http://software.broadinstitute.org/gsea/index.jsp) was used to assess the distribution of gene sets across the differential expression profile of ALS compared with Healthy myotubes. There were 6349 gene sets tested, including all of the Gene Ontology Biological Process and Cellular Component collections, and all of the Canonical Pathways from MSigDB. In addition, custom gene sets were created listing genes encoding the known protein binding partners of FUS^25^ and TDP43.^26^ Cytoscape v3 and the enrichment map plugin were used to create a graph representing as nodes the gene sets identified by GSEA to be significantly enriched with FDR < 0.05, with edges shown for those pairs of gene sets having overlap coefficient >0.5. Cytoscape's selection features were used to isolate a sub‐graph of the enrichment map showing only the FUS‐binding and TDP43‐binding gene sets and those gene ontology or canonical pathway gene sets with which they shared genes (overlap coefficient >0.5).

### Quantitative RT‐qPCR

- *cDNA synthesis*: RNA from 3 days differentiated muscle cells was extracted as described earlier. A 1 μg of RNA was used to synthesize cDNA using M‐MLV Reverse Transcriptase (LifeTechologies™). Quantitative PCR was performed on LightCycler® 480 Instrument (Roche) using LightCycler® 480 DNA SYBR Green I Master (Roche).
- *Housekeeping genes*: Beta‐2‐microglobulin (B2M) showed a constant expression level in all samples and was used to normalize the gene expression levels of extracellular vesicle markers.

**Table:** 

Primers		Sequence (5′ → 3′)
β2M	Fw Primer	CTCTCTTTCTGGCCTGGAGG
	Rev Primer	TGCTGGATGACGTGAGTAAACC

- *Extracellular vesicle markers and FUS expression level*: Quantitative PCR was performed on LightCycler® 480 Instrument (Roche) using LightCycler® 480 DNA SYBR Green I Master (Roche). Primers used are listed in table below. The amplification efficiency of the reaction was calculated using data from a standard curve using RT products from control cells as reference samples (1:20, 1:100, 1:500, and 1:2500 dilutions used). The gene expression level was estimated using the comparative Ct method—Ct representing the cycle at which the fluorescence signal crosses the threshold as previously described.^18^

**Table:** 

		Sequence (5′ → 3′)
Primers	Rev primer	AGGAAAAGCCAGGTCCGAAC
CD81	Fw primer	TTCCACGAGACGCTTGACTG
	Rev primer	CCCGAGGGACACAAATTGTTC
CD63	Fw primer	CAGTGGTCATCATCGCAGTG
	Rev primer	ATCGAAGCAGTGTGGTTGTTT
CD82	Fw primer	GCTCATTCGAGACTACAACAGC
	Rev primer	GTGACCTCAGGGCGATTCA
RAB5b	Fw Primer	TCACAGCTTAGCCCCCATGTA
	Rev primer	CTTCACCCATGTCTTTGCTCG
TSG101	Fw primer	GAGAGCCAGCTCAAGAAAATGG
	Rev primer	TGAGGTTCATTAGTTCCCTGGA
FUS	Fw primer	TGGTCTGGCTGGGTTACTTT
	Rev primer	TAACTGGTTGGCAGGTACGT

#### RNA staining

The ALS and healthy muscle cells were differentiated for 3 days before being fixed using 4% paraformaldehyde and permeabilized for 1 h at RT (5% BSA; 20% FBS; 0.5% Triton X100; 0.5% Tween20). Cells were then stained with a 20 μg/mL acridine orange solution for 10 min at RT.

#### Muscle cell lines overexpressing wild type forms of FUS, SOD1 and TDP43

Healthy human muscle cell lines were transduced with previously published plasmid coding either for a tagged form of wild‐type FUS (FUS‐3xFLAG,^27^), or wild type SOD1 (SOD1‐3xFLAG, modified WT‐SOD1 plasmid from^28^) or TDP43 (TDP43‐3xFLAG, modified WT‐TDP43‐YFP plasmid from Johnson *et al*.^29^) and a selection was done with hygromycin. The 50 000 Cont, FUS_FLAG_, TDP43_FLAG_, and SOD1_FLAG_ muscle cells were plated in 8 well Ibidi® plates in proliferative medium. The next day, myogenic differentiation was induced by replacing the medium with DMEM, and cells were treated with 0.5 μg of healthy and ALS MuVs. MuVs were applied for 3 days, before fresh DMEM medium was applied to the cultures. At 6 days of differentiation, cells were fixed and analysed for cell death and cell blebbing as described earlier. All cell cultures treated with MuV were compared and normalized to untreated cell cultures.

### Proteomic

- The MuV pellets were re‐suspended in 25 μL 8 M Urea, 50 mM ammonium bicarbonate, pH 8.5, and reduced with DTT for 1 h at 4°C. Protein concentrations were then quantified using Pierce BCA Protein Assay kit (ThermoFisher®). MuV proteins were kept at −80°C.
- *Proteome profile determined by Mass spectrometry*—20 μg of MuV protein was trypsin digested using a SmartDigest column (Thermo) for 2 h at 70°C and centrifuged at 1400 rpm. Peptides were then fractionated into eight fractions using a high pH reverse phase spin column (Thermo). Fractioned peptides were vacuum dried, resuspended, and analysed by data‐dependent mass spectrometry on a Q Exactive HF (Thermo) with the following parameters: Positive Polarity, m/z 400–2000 MS resolution 70 000, AGC 3e6, 100 ms IT, MS/MS resolution 17 500, AGC 5e5, 50 ms IT, Isolation width 3 m/z, and NCE 30, cycle count 15.
- *Database Search and Quantification*—The MS raw data sets were searched for protein identification for semi tryptic peptides against the Uniprot human database for semi tryptic peptides including common contaminants, using MaxQuant software (version 1.6.2.1) (https://www.biochem.mpg.de/5111795/maxquant). We used default parameters for the searches: mass tolerances were set at ±20 ppm for first peptide search and ±4.5 ppm for main peptide search, maximum two missed cleavage; and the peptide and resulting protein assignments were filtered based on a 1% protein false discovery rate (thus 99% confidence level). The 1254 proteins were detected in at least one sample. The mass spectrometry proteomics data have been deposited to the ProteomeXchange Consortium via the PRIDE partner repository with the dataset identifier PXD015736.

### Knockdown of FUS with siRNA

There were 100 000 cells plated in μ‐Slide 4 Well ibiTreat (Ibibi®) one day before differentiation was induced. On Day 2 of differentiation, cells were transfected using Lipofectamine RNAiMAX Reagent with 200 nM s5401 FUS siRNA (LifeTechnologies™) and treated with a low dose of PKH26‐labelled ALS MuVs. MuVs were integrated by the cells for 3 days before fresh differentiation medium was added to the 5 days differentiated cells. The cells were harvested at Day 8 of differentiation to check for RNA levels by RT‐qPCR and to perform cell death analysis, immunostaining for RPL5, and distribution analysis of RNA/DNA using acridine orange staining.

### Western Blotting

Extracted ALS and healthy vesicles were resuspended in lysis buffer (8 M urea; 2% SDS; 10 μL/mL protease inhibitor cocktail), and protein was extracted from muscle biopsies using RIPA buffer. Extracted proteins were loaded into NuPage polyacrylamide 4–12% BisTris gels for electrophoresis under reducing conditions (Calnexin, Flotillin, ALIX, FUS, SOD1, TDP43, RPL5, and skeletal alpha‐actin) and non‐reducing conditions (CD63 and CD81). Transfer on polyvinylidene difluoride (PVDF) membrane was performed using the iBlot® Dry Blotting System (Life Technologies™) and upon transfer polyacrylamide gels were stained with Blue Coomassie Gel Code Blue Stain Reagent (LifeTechnologies™) to visualize proteins. Immunoblotting was carried out using the iBind™ Flex Western System and primary antibodies (refer to *Table* S2) with respective secondary antibodies (Goat anti‐Mouse HRP, Donkey anti‐Goat HRP, Goat anti‐Rabbit HRP). The signal was detected using the Amersham ECL™ Prime Western blotting Detection Reagent and the UVP ChemiDoc‐It2 Imager.

### Statistics

All values are presented as means ± SEM. Student's *T* test was used to compare differences between ALS and control samples for all the protein quantifications, electron microscopy quantifications, MuV integration, immunostaining (FUS, Acridine Orange, RPL5, Caprin1), FUS expression level, MuV‐treated MN and myotube death, and myotube atrophy. A Kolmogorov–Smirnov test was used to compare the distribution of number of vesicles per MVBs in ALS and healthy myotubes, the distribution of neurites branching in ALS and healthy MuV‐treated MN, the distribution of H2AX expression levels in myonuclei treated with different doses of MuVs, and the distribution of myotube nuclear numbers in control myotubes treated with ALS and control MuVs. One‐way ANOVA followed by a Tukey's multiple comparison test was used to evaluate the differences in the in silico secretome, in the immunostaining for extracellular vesicle markers, RT‐qPCR, neurites length, and MN treatments. Two‐way ANOVA followed by Bonferroni post‐hoc test was used to evaluate dose response of MuV on MN survival, changes in bleb counts, and cell death in different cells lines (Cont, FUS_FLAG_, SOD1_FLAG_, and TDP43_FLAG_). Enrichment testing *P* values were determined by the Fisher's exact test (for enrichment testing of custom lists of FUS‐binding and TDP43‐binding proteins) or represent a multiple‐testing adjusted value based on deviation from expected rank as calculated by the EnrichR tool (for enrichment testing across all GO Molecular Functions). Differences were considered to be statistically different at *P* < 0.05.

## Results

### Amyotrophic lateral sclerosis patient muscle cells accumulate vesicles

**Figure 1:**
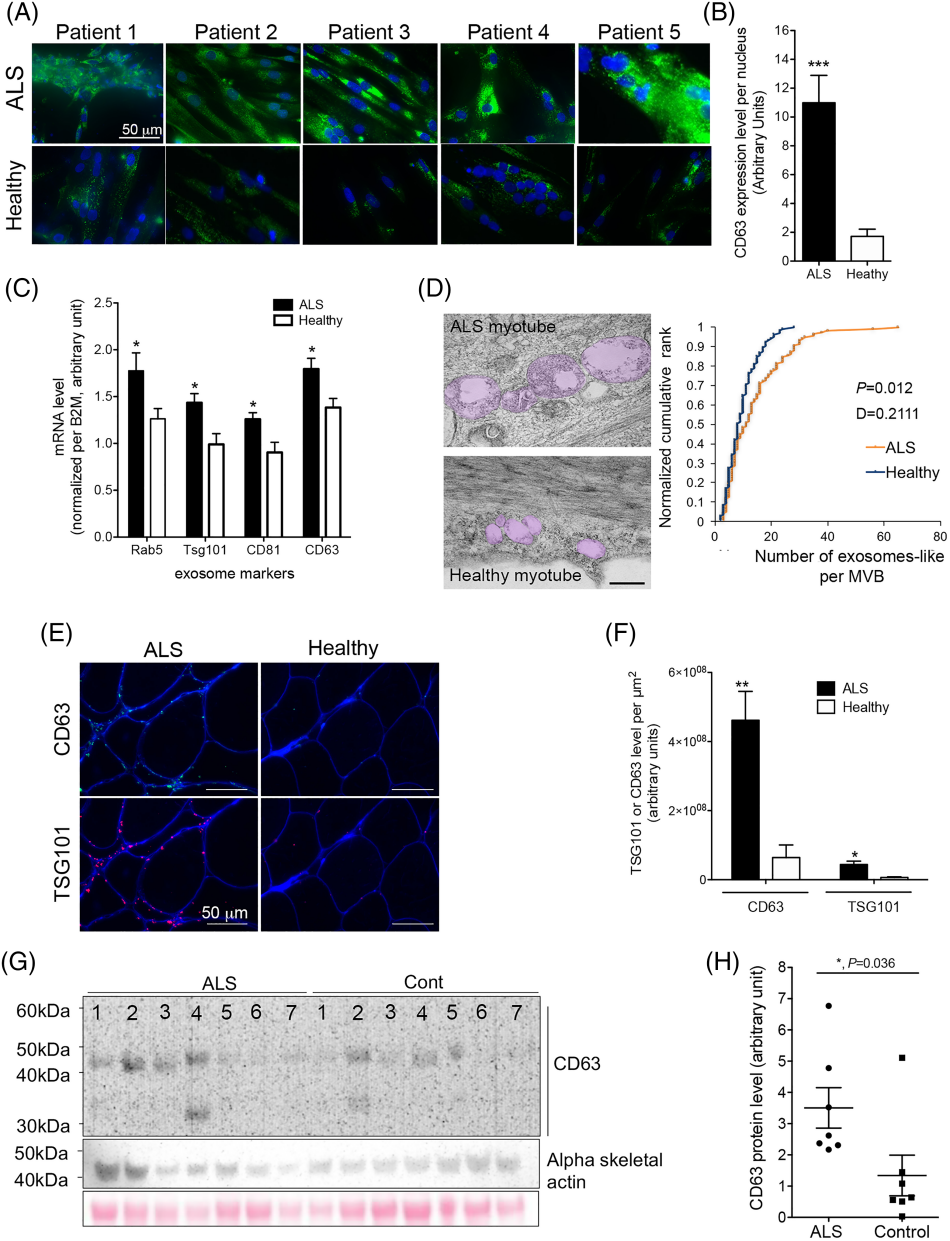
Accumulation of extracellular vesicle markers in myotubes and muscle of amyotrophic lateral sclerosis (ALS) patients. (A) Panel showing representative images of CD63 immunostaining performed on cultured myotubes from different patients. (B) Quantification of CD63 fluorescence signal normalized per myonucleus (70–108 myonuclei were analysed per individual, with *n* = 5 ALS and 6 healthy subjects). *** significantly different from healthy with *P* < 0.001. (C) mRNA encoding for extracellular vesicle proteins normalized per B2M mRNA level are upregulated in ALS myotubes compared with healthy (*n* = 7 ALS, 6 healthy). * significantly different from healthy with *P* < 0.05. (D) Multi‐vesicular bodies that are present in ALS myotubes contain more exosome‐like vesicles than those of healthy controls. Left panel: Representative electron micrographs showing an accumulation of exosome‐like vesicles in multi‐vesicular bodies (MVBs; highlighted in pink—shown without highlight in *Figure* S1A). Scale bar = 500 nm. Right panel: quantification of the number of exosome‐like vesicles in the MVBs (100 MVBs per condition were analysed). Two sample Kolmogorov–Smirnov test confirmed the visual impression that the MVBs of ALS muscle contain a greater number of exosome‐like vesicles compared with healthy controls (*P* < 0.05). (E) Representative images of extracellular vesicle markers CD63 and TSG101 immunostaining in ALS and healthy muscle. CD63 green, TSG101 red, and dystrophin blue. Scale bar 50 mm. (F) Quantification of extracellular vesicle markers CD63 and TSG101 in muscle biopsies (Pixel per square millimetre), *n* = 6 ALS, 5 healthy for CD63, and *n* = 4 per group for TSG101; * and ** significantly different from healthy subjects, *P* < 0.05 and *P* < 0.01, respectively. (G) Western blots showing the expression of CD63 protein in muscle biopsies from ALS and healthy subjects (*n* = 7 per group). Skeletal alpha actin and Ponceau staining on the membrane are shown as loading controls. (H) Quantification of CD63 protein level normalized per loading control (*n* = 7 per group). * significantly different from healthy with *P* < 0.05. Values are means ± SEM. Refer also to *Figure* S1.

To investigate the role of vesicles secreted by ALS muscle cells, myoblasts were extracted from biopsies of confirmed ALS patients at an early stage of pathology (refer to *Table* 1 for patient description, and a breakdown of which samples were used in which experiment). Among the 27 sporadic ALS subjects, genetic screening identified only four with known ALS‐causative mutations (three carrying the *C9orf72* hexanucleotide repeat expansion, one with aberrant *ATXN2* repeat length). A consistent accumulation of extracellular vesicle markers CD63 (*Figure* 1A,B) and TSG101 (*Figure* S1B) in ALS myotubes was observed by immunostaining. Extracellular vesicle markers were higher by RT‐qPCR (*Figure* 1C), and multivesicular bodies filled with exosome‐like vesicles were observed by electron microscopy (*Figures* 1D and S1A). *In vivo*, ALS patient muscle biopsies presented an increased frequency of multi‐vesicular bodies (MVBs) (0.017 MVBs/sarcomere in ALS muscles, vs. 0.004 MVBs/sarcomere in healthy controls; *Figure* S1C,D), an accumulation of extracellular vesicle markers at the periphery of the myofibres (*Figure* 1E,F), and an increased expression level of the vesicle marker CD63 (*Figure* 1G,H).

### Secretion of amyotrophic lateral sclerosis patient muscle cell vesicles

**Figure 2:**
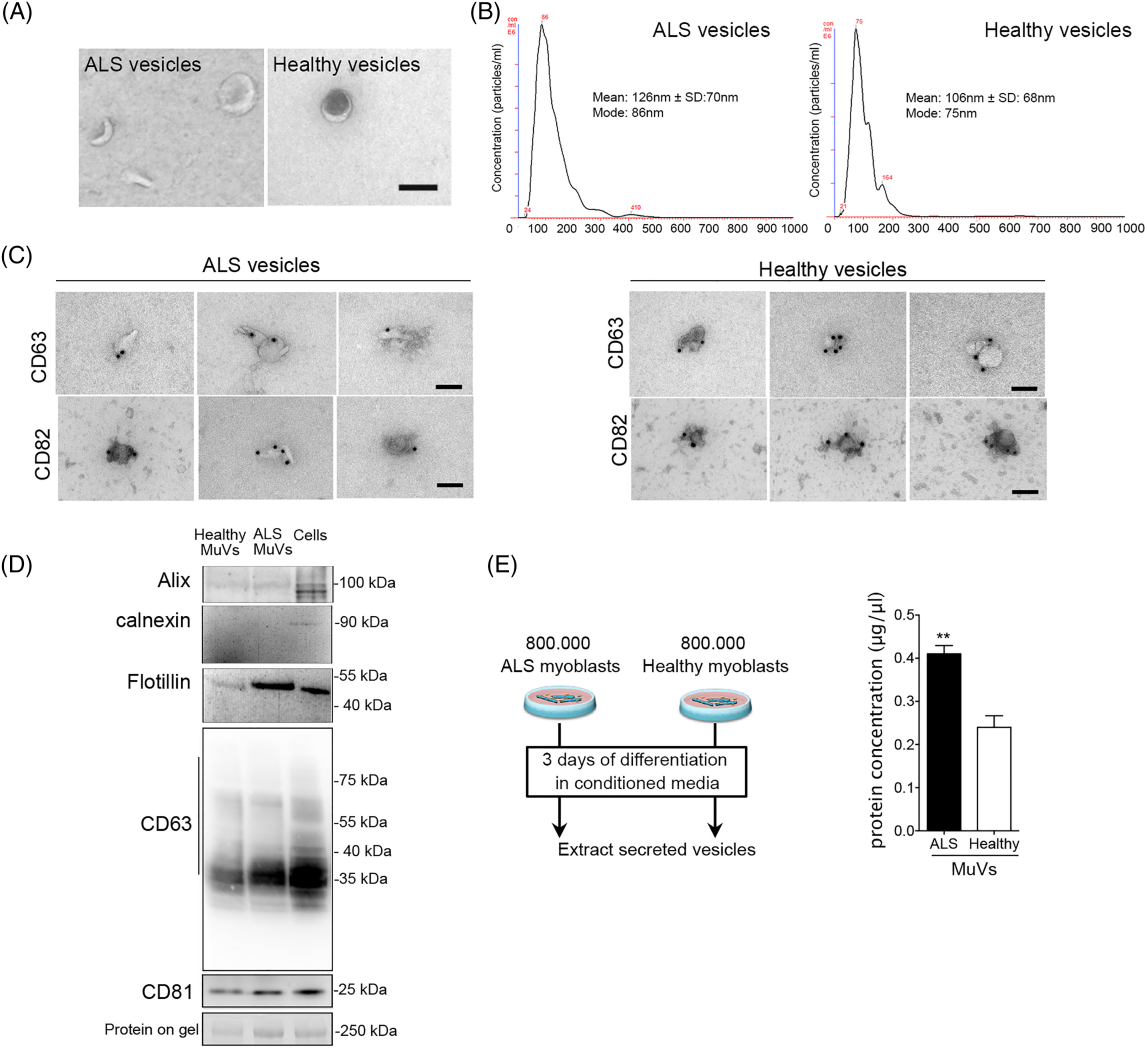
Increased accumulation and secretion of vesicles by amyotrophic lateral sclerosis (ALS) myotubes. (A) Representative electron micrographs of vesicles extracted from the culture medium of ALS or healthy myotubes. Scale bar = 100 nm. The extracted vesicles have the typical cup‐shape of exosomes. (B) NanoSight analysis showing that ALS and healthy vesicle sizes range between 90–200 nm. (C) Representative electron micrographs of vesicle immunostaining showing that both ALS and healthy muscle vesicles (MuVs) express CD63 and CD82. bar = 100 nm. (D) ALS and healthy muscle vesicles are positive for Alix, flotillin, CD63 and CD81, and negative for calnexin. (E) The MuV fraction secreted by ALS myotubes contained 1.7 more protein than healthy MuV fraction. Left panel: schema summarizing the experimental procedure. Briefly, the same number of ALS and healthy myoblasts were differentiated, and after 3 days, the culture medium was harvested to extract the MuVs as described in material and methods. Right panel: protein quantification of MuVs; 800 000 differentiated myoblasts per subject, with *n* = 4 subjects per group. ** significantly different from healthy myotubes (*P* < 0.01). Values are means ± SEM. Refer also to *Figure* S2.

Importantly, because primary muscle stem cells have a limited number of divisions (~30), all experiments were carried out before the cells reached 21 divisions, thereby avoiding excessive population doublings which could lead to senescence and experimental artefacts.^18^, ^20^, ^30^ In both ALS and healthy vesicle extracts, exosome‐like vesicles with a typical cup‐shaped morphology (*Figure* 2A) and sizes ranging from 90 to 200 nm (*Figure* 2A–C) were observed. The muscle vesicles were positive for exosomal markers such as CD63, CD82, CD81, Flotillin, and ALI, and were negative for calnexin, an endoplasmic reticulum marker normally absent in the exosome fraction (*Figure* 2C,D). In addition, the vesicle extracts were free of albumin and other contaminants (*Figure* S2B, and proteomic data GSE122261). The MuV fraction secreted by ALS myotubes contained 1.7‐fold more protein than that secreted by an equal number of healthy control myotubes (*Figures* 2E and S2B).

### Secreted amyotrophic lateral sclerosis vesicles are neurotoxic

**Figure 3:**
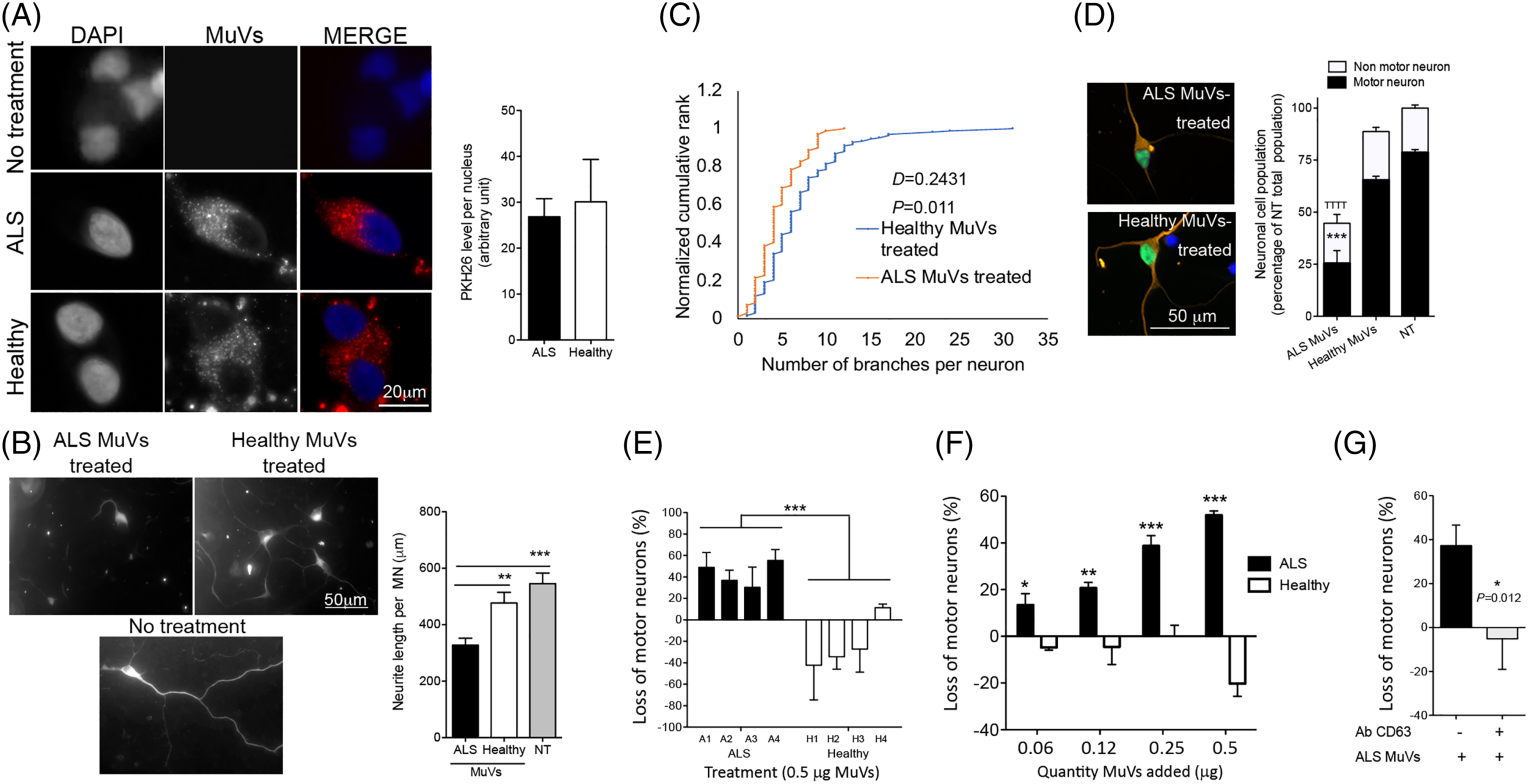
Amyotrophic lateral sclerosis (ALS) muscle vesicles induce decreased neurite length and branching, and increased death, of human induced pluripotent stem cells derived motor neurons. (A) Equal quantities of ALS and healthy muscle vesicles (MuVs) are taken up by motor neurons. Red: MuVs labelled with PKH26 and added to the culture medium of iPSC‐derived motor neurons. (B) Neurite lengths of motor neurons are shortened when treated with ALS MuVs compared with healthy MuVs. Left panel: representative images of MuV‐treated motor neurons. Right panel: quantification of neurite length (8 to 15 motor neurons analysed per well, with *n* = 5 wells per treatment). ** and *** significantly different from ALS values *P* < 0.01 and *P* < 0.001, respectively. (C) Neurites of iPSC‐motor neuron (MN) cells have fewer neurite branch‐points following treatment with ALS MuVs compared with treatment with healthy MuVs; two‐sample Kolmogorov–Smirnov test confirmed the visual impression that the number of branches per neurite is decreased (*P* = 0.011). (D) Neurotoxicity of ALS MuVs is specific to motor neurons. Right panel: representative images of human iPSC‐MN treated with ALS or healthy MuVs. iPSC‐MN are positive for motor neuron marker Islet1/2 (green) and neuronal marker Tuj1 (orange). Right panel: quantification of human iPSC neuron and MN cells (10 frames per well, *n* = 3 wells per condition). ***P < 0.001 and ^TTT^ *P* < 0.001, significantly different from healthy‐MuV‐treated and non‐treated cells, respectively. (E) Quantification of the death of human iPSC‐motor neurons treated with equal amounts (by protein content: 0.5 μg) of ALS or healthy MuVs. MuVs were extracted from sporadic ALS patients negative for known mutations (each bar represents a different subject: *n* = 4 subjects per group, each in triplicate). ***P < 0.001, significantly different from healthy values. (F) Motor neuron survival in response to increasing concentrations of muscle vesicles from the muscle cells of ALS or healthy subjects. MuVs from the muscle cells of ALS patients (black bars) or healthy subjects (white bars) were added to cultures of iPSC‐derived MN at concentrations of 0.06, 0.12, 0.25, or 0.5 μg/150 μL. The total number of neurons at 72 h after treatment was counted in each condition and expressed as a percentage of the cell death that was observed in cultures of untreated motor neurons at the same time‐point. ANOVA 2‐factor followed by Bonferroni post‐hoc test was performed. *P < 0.05, **P < 0.01, and ***P < 0.001, significantly different from healthy‐MuV‐treated at that concentration. (G) Pre‐treatment of the ALS MuVs with CD63 antibody significantly decreased iPSC‐motor neuron (MN) cell death. * significantly different from ALS values, *P* < 0.05. Values are means ± SEM. Refer also to *Figure* S3.

To test neurotoxicity, 0.5 μg of ALS or healthy MuVs were added to the culture medium of healthy human iPSC‐derived motor neurons (hiPSC‐MN). Because ALS myotubes secrete more MuVs than healthy myotubes, this quantity corresponded to 2 ALS myonuclei or 3.7 healthy myonuclei per MN. Following uptake of MuVs (*Figure* 3A), only ALS MuVs and not healthy MuVs resulted in shorter neurites (*Figure* 3B), with less branching (*Figure* 3C) and a greater cell death (*Figure* 3D,E) 72 h post‐treatment. Cell death as a result of ALS MuVs was consistent across multiple patients (*Figure* 3E) and showed a dose response when different quantities of ALS MuV protein were loaded—ALS MuV toxicity was significant at all concentrations (*Figure* 3F). Conversely, when MuV uptake was decreased by preincubating MuVs with CD63 antibody (*Figure* S3A), hiPSC‐MN death was dramatically decreased (*Figure* 3G). Similarly, when added to the culture medium of healthy human myotubes, ALS MuVs induced myotube atrophy (*Figure* S3B), and cell stress (*Figure* S3C) leading to cell death (*Figure* S3D–F). The quantity of cell death was decreased when less MuV protein was loaded, though ALS MuV toxicity remained greater than healthy MuV toxicity at either dose (*Figure* S3F), suggesting that toxicity is dependent on both the quantity and content of MuVs.

### Amyotrophic lateral sclerosis muscle vesicles are enriched in proteins involved in RNA processing

**Figure 4:**
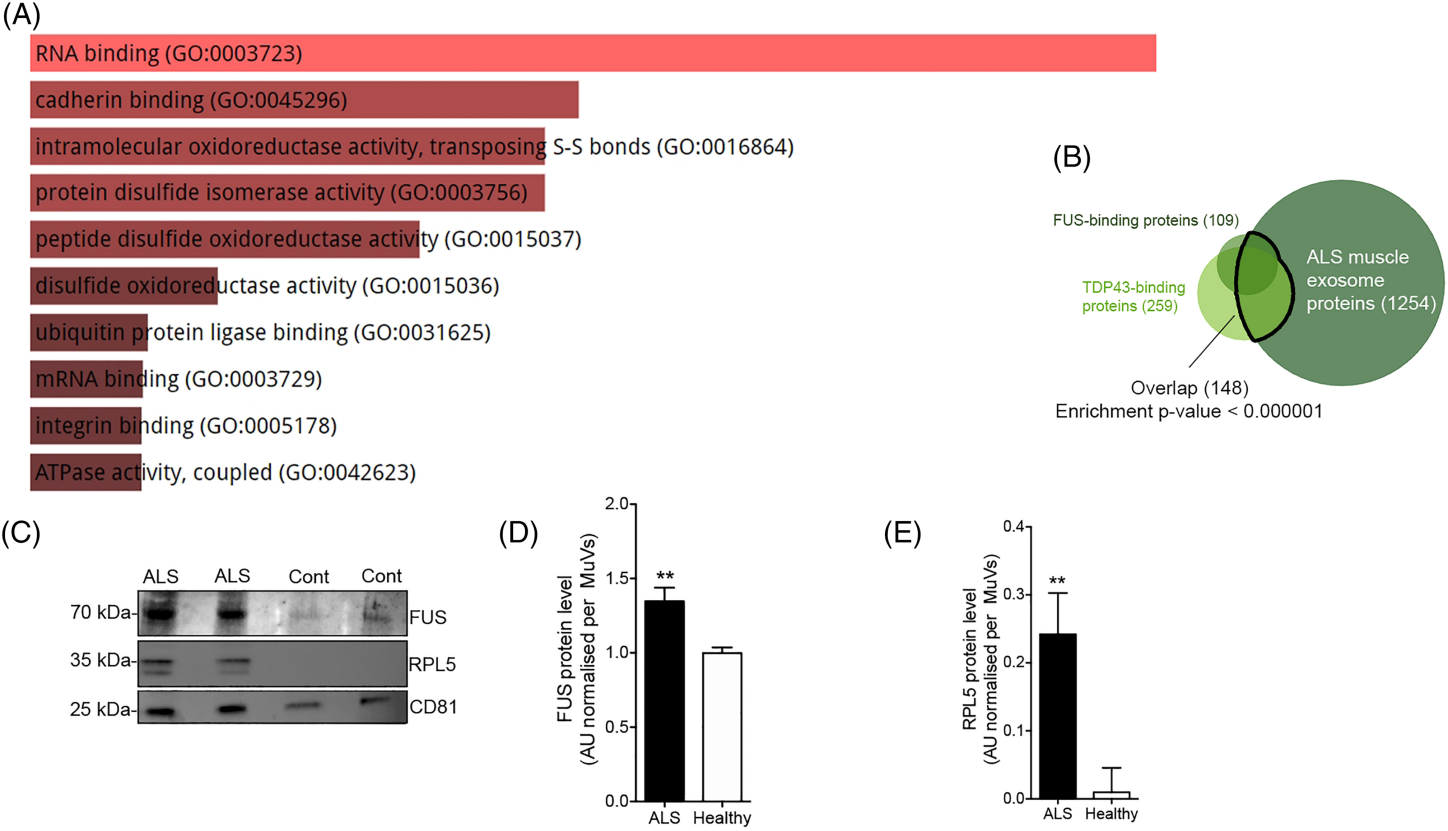
The proteomic content of muscle vesicles is enriched for FUS‐binding and TDP43‐binding proteins.(A) Output of EnrichR tool showing relative enrichment scores of gene ontology molecular functions among peptides that were observed at consistently higher levels in the muscle vesicles (MuVs) of amyotrophic lateral sclerosis (ALS) subjects compared with healthy controls (FDR *P* value for the RNA‐binding GO term was <1 × 10^−7^). (B) Whereas only 1.5% of all proteins are known binding partners of FUS and/or TDP43, they represent 13.4% (148 of 1254) of the proteins detected by proteomic analysis of ALS MuVs contents (Fisher's test *P* < 0.000001). (C) Representative images of Western blot showing the presence of FUS and RPL5 in muscle vesicles. CD81, extracellular vesicle markers. (D) FUS protein level in higher in ALS MuVs. Quantification by Western blot of FUS level in MuVs. **P < 0.01, significantly different from healthy values. *n* = 15 ALS and 13 healthy. (E) RPL5 protein level in higher in ALS MuVs. Quantification by Western blot of RPL5 level in MuVs. *P < 0.05, significantly different from healthy values. *n* = 6 subjects per group. Values are means ± SEM. Refer also to *Figure* S4, *Tables* S1 and S2.

Proteomic analysis of vesicle content revealed that, of 53 peptides observed at consistently higher levels in the MuVs of ALS subjects compared with healthy controls, 21 were annotated to the RNA‐binding molecular function (*Figure* 4A; enrichment FDR *P* < 1 × 10^−7^; *Table* S1). Similarly, of 453 proteins detected only in ALS and not in healthy controls, 87 were involved in RNA‐binding (enrichment FDR *P* < 1 × 10^−14^; *Table* S2). ALS MuVs contained many known protein binding partners of the RNA‐processing proteins FUS^25^ and TDP43^26^ (enrichment *P* < 1 × 10^−6^; *Figure* 4B), with 58% (64 of 109) of FUS binding partners being detected, along with FUS itself. Similar enrichment (*P* from 0.001 to <1 × 10^−6^) was observed against other protein lists of FUS‐binding and TDP43‐binding partners.^31^, ^32^ FUS and RPL5—a FUS binding partner—were at a higher level in ALS MuVs by Western blotting (*Figure* 4C–E). Interestingly, neither SOD1 nor TDP43 were detectable in human MuVs (*Figure* S4).

**Figure 5:**
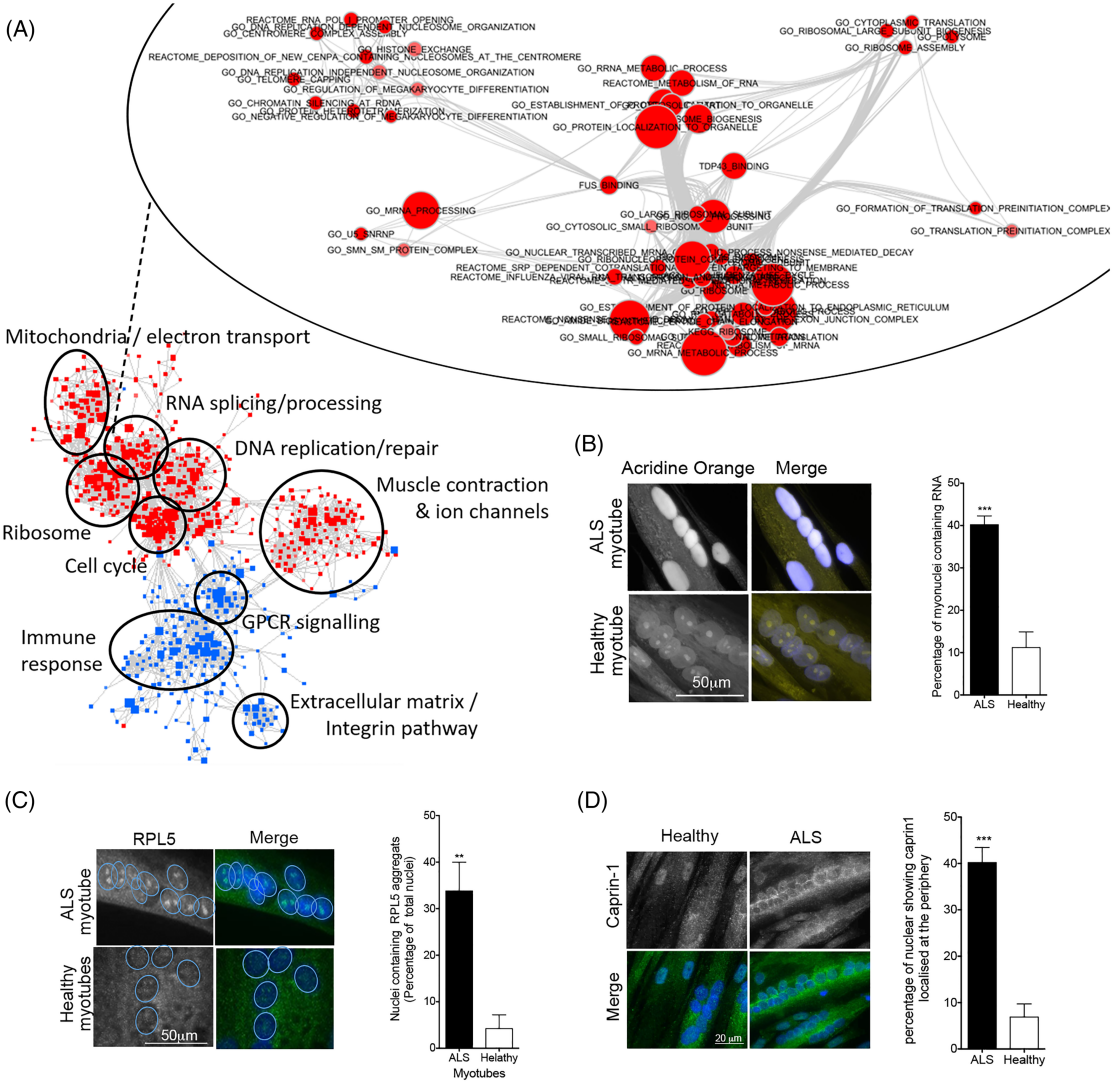
RNA processing is disrupted in amyotrophic lateral sclerosis (ALS) myotubes. (A) Enrichment map representing overlap and clustering of the cellular processes and pathways for which gene expression is dysregulated in ALS myotubes compared with healthy controls (red = upregulated; blue = downregulated). Upper inset: enrichment map showing that genes encoding FUS‐binding and TDP43‐binding proteins are upregulated in ALS myotubes compared with healthy controls, and that these genes are shared with many RNA‐processing pathways that are similarly upregulated (red = upregulated). (B) ALS myotubes present an accumulation of RNA in their nuclei. Left panel: representative images of RNA localization in ALS and healthy myotubes. Right panel: percentage of myonuclei with high levels of RNA, assayed by acridine orange staining (50 myonuclei analysed per subject, with *n* = 5 subjects per group). ***P < 0.001 significantly different from healthy myotubes. (C) RPL5, a protein involved in RNA transport and stress granules, is granulated in ALS myonuclei. Left panel: representative images of RPL5 localization in ALS and healthy myotubes. Right panel: percentage of myonuclei with RPL5 granules (at least 500 nuclei analysed per subject, with *n* = 4 subjects per group). **P < 0.01 significantly different from healthy myotubes. (D) ALS MuVs induce an accumulation of RNA in motor neuron (MN) nuclei. Left panel: representative images of RNA localization in MN nuclei. Right panel: percentage of nuclei with accumulations of RNA (150 to 260 nuclei analysed per well, with *n* = 3 wells per condition). ***P < 0.001 significantly different from healthy myotubes. Values are means ± SEM.

Transcriptomic analysis of cultured myotubes suggested that genes encoding FUS‐binding and TDP43‐binding proteins were upregulated in the myotubes of ALS patients and that these genes were shared with many RNA‐processing pathways that were similarly upregulated (*Figure* 5A). ALS myotubes presented a greater nuclear accumulation of RNA (*Figure* 5B), and mislocalization of two FUS protein binding partners, RPL5 and caprin 1, that are involved in RNA processing and stress granule formation (*Figure* 5C,D).^25^

### Secreted amyotrophic lateral sclerosis vesicles affect RNA processing in recipient motor neurons

**Figure 6:**
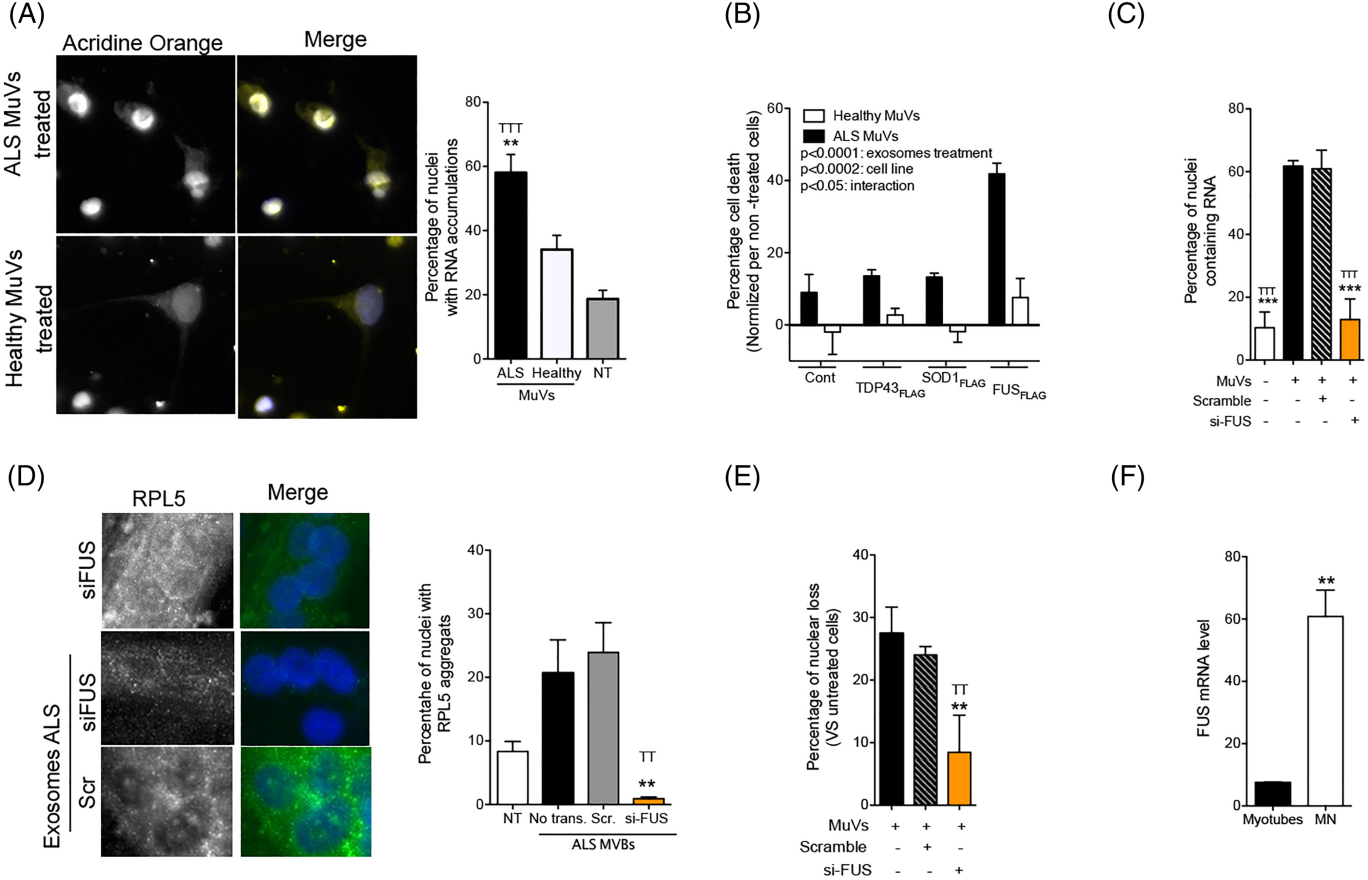
The toxicity of amyotrophic lateral sclerosis (ALS) muscle vesicles to healthy motor neurons involves the FUS protein and RNA processing. (A) ALS muscle vesicles induce an accumulation of RNA in motor neuron (MN) nuclei. Left panel: representative images of RNA localization in motor neuron (MN) nuclei. Right panel: percentage of nuclei with accumulations of RNA (150 to 260 nuclei analysed per well, with *n* = 3 wells per condition). **P < 0.01 and ^TTT^ *P* < 0.001 significantly different from MN treated with healthy MuVs and untreated MN, respectively. (B) Cell death induced by 0.5 μg of ALS MuVs is exacerbated in the presence of over‐expression of FUS. ANOVA 2 factors was performed, showing the effect of ALS MuVs (*P* < 0.0001), the effect varying due to cell line (*P* < 0.0002), and an interaction between the two parameters (*P* < 0.05) (10 frames per well analysed, with *n* = 3 wells per condition). (C) Percentage of nuclei with RNA accumulation are decreased when ALS MuVs are added to the culture medium of cells deficient for FUS (FUS expression level was reduced by 79.5% ± 4.2% with siRNA strategy, 10 frames per well analysed, with *n* = 3 wells per condition). ***P < 0.001 ALS MuV‐treated cells. ^TTT^ *P* < 0.001 ALS MuV‐treated scramble‐RNA cells, respectively. (D) RPL5‐aggregates are no longer observed following ALS‐MuV treatment when the recipient cells do not express FUS. Left panel: representative images of myotubes treated with siFUS or siScrambled control, and with addition or not of ALS MuVs. Myotubes are immunostained for RPL5 (green). Right panel: Percentage of myotube nuclei with RPL5 aggregates in untreated (‘No exo.’), ALS‐MuV‐treated with no knockdown (‘No trans’), ALS‐MuV‐treated with siScrambled knockdown (‘Scr.’), or ALS‐MuV‐treated with siFUS knockdown (‘si‐FUS’). Eight hundred to 1000 nuclei were analysed per well, with *n* = 3 wells per condition. ANOVA 1 factor was performed, showing the effect of ALS MuVs on the two different knock‐down conditions, with ** and ^TT^ representing significant difference from ‘No trans.’ and ‘Scr.’, respectively, *P* < 0.01. (E) Percentage of cell death is decreased when ALS MuVs are added to the culture medium of cells deficient for FUS (FUS expression level was reduced by 79.5% ± 4.2% with siRNA strategy, 10 frames per well analysed, with *n* = 3 wells per condition). **P < 0.01 ALS MuV‐treated cells, respectively. ^TT^ *P* < 0.01 ALS MuV‐treated scramble‐RNA cells respectively. (F) FUS mRNA level is significantly higher in MN compared with myotubes. **P < 0.01 significantly different from human myotubes (*n* = 3 per group). Values are means ± SEM. Refer to *Figure* S5.

Because a leading theory is that disruption of RNA metabolism—including RNA translation, transport, storage, and degradation—contributes to ALS physiopathology by affecting neuronal function and viability,^33^ and based on the results described earlier, we hypothesized an involvement of RNA processing in ALS MuV toxicity. When human iPSC‐MNs derived from healthy subjects were treated with ALS MuVs, RNA accumulated in their nuclei (*Figure* 6A), which has been reported to induce cell death.^34^ Similar results were obtained when ALS MuVs were added to the cultures of human myotubes from healthy subjects (*Figure* S5A). We hypothesized that ALS MuV toxicity in recipient cells may be mediated through the FUS pathway. To test this, 0.5 μg of ALS or healthy MuVs were added to the culture medium of a human muscle cell line over‐expressing a tagged form of wild‐type FUS (FUS‐_FLAG_; previously published^27^). This induced an increase in cell death from 8% to 42% (*Figure* 6B), accompanied with greater cellular stress (*Figure* S5B)—the same was not observed in cell lines that over‐expressed tagged forms of wild‐type TDP43 and SOD1 (*Figures* 6B and S5B). Conversely, when ALS MuVs were added to the culture medium of cells in which FUS was knocked down, lower proportions of nuclei with accumulated RNA (*Figure* 6C) and RPL5 granules (*Figure* 6D) were observed, and the quantity of cell death was reduced (*Figure* 6E). These data implicate the FUS pathway in MuV toxicity and suggest that increased levels of FUS heighten sensitivity to this toxicity, while lower levels reduce it. We note that, relative to muscle cells, high levels of FUS are observed in hiPSC‐MN (*Figure* 6F).

## Discussion

Extracellular vesicles are suspected to carry toxic elements from astrocytes towards motor neurons,^7^ and pathological proteins have recently been identified in circulating extracellular vesicles of sALS patients.^35^ Here, ALS muscle vesicles are shown to be toxic to motor neurons, which establishes the skeletal muscle as a potential source of vesicle‐mediated toxicity in ALS.

The accumulation and over‐secretion of muscle vesicles was observed as a consistent feature of sporadic ALS patients in this cohort, including patients carrying mutations in *C9orf72* or *ATXN2*, suggesting that this may be a common feature across many or all sporadic and familial forms of ALS. The consistent observation of a pronounced RNA processing and protein mislocalization phenotype in the ALS myotubes suggests that these cells recapitulate aspects of the disease mechanism that have been observed in motor neurons.

The observation that ALS MuVs act on RNA transport, that overexpression of wild‐type FUS in MuV‐recipient cells resulted in increased recipient cell death, and that RNA transport protein mislocalization is partially corrected and cell death reduced when FUS expression was knocked down in recipient cells, is consistent with a body of literature suggesting an RNA processing blockade mechanism in ALS MN.^36^, ^37^ As shown, FUS expression is relatively high in iPSC MN compared with muscle cells (*Figure* 6F), and we also note that the cerebral cortex is among the tissues with the highest reported levels of FUS mRNA (http://www.proteinatlas.org ^38^). While here we examined the relationship of FUS to ALS MuV toxicity in recipient muscle cells, further investigation, focused on MN, will be necessary to understand the mechanistic interplay of FUS and RNA‐processing with muscle vesicle contents and the potential role in ALS pathology.

The observation that FUS and many of its binding partners are present in MuVs is of interest in the context of recent observations that normal function of FUS is required for normal neuromuscular junction development in mice, and that co‐culture of iPSC‐derived motor neurons with myotubes from FUS mutated patients resulted in impaired endplate maturation, which was proposed to be due to intrinsic FUS toxicity in both muscle and MN.^39^ Similar experiments, not only on the cells of patients with FUS mutations but also on sporadic ALS more generally, may help to explore the relationship of vesicle‐mediated toxicity to the neuromuscular junction.

Several papers have described a muscle phenotype in ALS that occurs independently and prior to muscle denervation, such as metabolic imbalance,^40^, ^41^ oxidative stress,^42^ and mitochondrial dysfunction.^43^ However, the role of muscle in ALS is unresolved. While an ALS‐like phenotype was observed in mice when exogenous human mutant SOD1 expression was restricted to the skeletal muscle,^42^, ^44^ other studies knocking down mutant SOD1 expression in skeletal murine muscle did not show any significant decreases in the progression of symptoms.^45^, ^46^ The differences observed between these studies reveal the difficulties to assess the role of muscle in ALS, as the success of targeting whole skeletal muscle requires intravenous injection of a large amount of adeno‐associated virus particles.^47^ The secretion of muscle cell vesicles that are toxic towards motor neurons *in vitro* could suggest a potential role of muscle in ALS pathology. However, further *in vivo* experiments in appropriate animal models are required, to test the capacity of MuVs to diffuse *in vivo*, and their capacity to cross the blood brain barrier or to be absorbed directly at the neuromuscular junction.

## Conflict of interest

The authors declare that they have no conflict of interest.

## Supporting information


**Figure S** **1:** Accumulation of exosomal markers in myotubes and muscle of ALS patients.
(A) Representative electron micrographs showing an accumulation of exosome‐like vesicles in multi‐vesicular bodies (Black arrows indicate MVBs). Bar = 500 nm. Lower panels: closer crop of the upper panel frames. (B) Accumulation of TSG101 in ALS human myotubes. Left panel: representative images of TSG101 immunostaining performed on cultured myotubes from different patients. Right panel: Quantification of TSG101 fluorescence signal normalized per myonucleus (*n* = 5 ALS and 6 healthy subjects). *, significantly different from healthy with *P* < 0.05. (C) Quantification of multi‐vesicular bodies per sarcomeres that are present in ALS and healthy muscle biopsies. *n* = 3 ALS and 3 healthy subjects). *, significantly different from healthy with P < 0.05. (D) Representative electron micrograph of sALS muscle longitudinal section. Multivesicular body (MVB) is highlighted in purple. The MVB contain exosome‐like vesicles.
**Figure S2:** More vesicles are secreted by ALS than by healthy myotubes
(A) Muscle vesicle extraction protocol adapted for primary muscle stem cells. Conditioned media from differentiated myoblasts were cleared of cell debris, apoptotic bodies and microparticles through sequential centrifugations and filtration. Muscle vesicles were then precipitated using exosome isolation reagent and washed with PBS using 100 k MWCO column. Rinsed MuVs were then either used for proteomic analysis and western blot, or labelled and added to the culture medium of targeted cells. (B) SDS‐PAGE analysis showing a greater quantity of protein in muscle vesicles enriched fraction secreted by 800,000 ALS myotubes compared to healthy controls. Left panel: representative image of SDS‐PAGE. Right panel: quantification of protein levels per well. *, significantly different from healthy, *P* < 0.05.
**Figure S3:** ALS muscle vesicles are toxic toward healthy human myotubes.
(A) Pre‐treated the MuVs with CD63 antibody significantly decrease their uptake iPSC‐MN. Left panel: representative images showing the ALS MuVs uptake by iPSC‐MN. MuVs were labelled with PKH26. Right panel: quantification of PKH26 labelled MuVs on recipient cells. ***, *P* < 0.001 versus non‐CD63 pre‐treated. (B) ALS MuVs induce muscle cell atrophy. Left panel: Representative images of healthy myotubes treated with ALS or Control MuVs. Myotubes are stained with myosin heavy chain (green). Right panel: quantification of myonuclear domain size (area of myosin heavy chain staining divided by the number of nuclei). The ALS‐MuVs treated myotubes have a smaller myonuclear domain. * *P* < 0.05, significantly different from healthy values. 2,000 to 4,000 myonuclei were analysed per subject, with *n* = 3 subjects per group. (C) ALS MuVs induce cell stress. Counts of blebs per healthy myotube at different time‐points after treatment with ALS or control MuVs. Five myotubes per time point per subject were analysed, with *n* = 3 subjects per group. ANOVA 2 factor interaction, time and treatment *P* < 0.001. (D) Levels of cell death marker H2Ax are increased in myonuclei of ALS‐MuVs‐treated myotubes compared to Healthy‐MuVs‐treated myotubes. Two hundred to 400 myonuclei per subject were analysed, with *n* = 3 subjects per group. * *P* < 0.05, significantly different from healthy values. (E) A greater loss of myonuclei is observed in cultures treated with ALS MuVs compared to healthy MuVs. n = 3 subjects per group. ** *P* < 0.01, significantly different from healthy controls. (F) Decreasing ALS MuVs quantity leads to decreased cell death. Left panel: representative immunostaining of H2‐Ax immunostaining of healthy myotubes treated with ALS or control MuVs. Right panel: The graph represents a distribution of H2Ax level in myonuclei treated with either 4 or 8 mg of ALS MuVs (curves in orange and red), or with 4 or 8 mg of healthy MuVs (curves in light and dark blue). One hundred and eighty to 300 nuclei were analysed per subject and per condition, with *n* = 3 per subject. Two factor ANOVA was performed, showing the effect of ALS MuVs (*P* < 0.0001), the effect varying due to quantity (*P* = 0.0110), and an interaction between the two parameters (*P* = 0.0301).
**Figure S4:** Representative western blot showing the absence of detection of SOD1 and TDP43 in MuVs.
**Figure S5:** ALS MuVs affect RNA transport in healthy myotubes and induce cell stress.
(A) Percentage of myonuclei positive for accumulation of RNA in myotubes treated with ALS or healthy MuV. Accumulation of RNA is increased in the myonuclei of ALS‐MuVs‐treated myotubes. *** *P* < 0.001, significantly different from healthy values. Seventy to 100 myonuclei per subject were analysed, with *n* = 4 subjects per group. (B) Cell stress induced by ALS MuVs is exacerbated in presence of over‐expression of FUS. ANOVA 2 factors was performed, showing the effect of ALS MuVs (*P* < 0.0001), the effect varying due to cell line (*P* < 0.005), and an interaction between the two parameters (P < 0.005).

**Table S1:** Table showing counts of peptides that were observed at consistently higher
levels in exosomes of ALS myotubes compared to healthy controls.
Peptides were filtered first to keep those for which the lowest value observed in ALS was greater than the highest value observed in controls, then were ranked by the difference in median values between ALS and controls.
**Table S2:** Table showing counts of peptides that were observed in at least one ALS muscle exosome sample but not in any healthy controls.
